# W-Compound can be used as a Biomarker for Fetal Thyroid Function and a Potential Tool for Screening Congenital Hypothyroidism

**Published:** 2022-06-30

**Authors:** Sing-Yung Wu, Haibo Zhao, Bi-Xin Xi, Dong-Bao Chen, Maria E. Fucito

**Affiliations:** 1 Department of Research, Veteran Administration University of California Irvine Medical Center, Long Beach, CA 90822.; 2 Analytical Pharmacology Core Facility, City of Hope, Duarte, CA 91010.; 3 Department of Obstetrics and Gynecology, University of California Irvine, Irvine, CA 92697.

**Keywords:** congenital hypothyroidism, fetal thyroid function, W-Compound, biomarker, neonatal screening

## Abstract

Sulfoconjugation is the major pathway for thyroid hormone (TH) metabolism, converting T4 to inactive metabolites, T4S, rT3S, and T3S in fetus, via sulfotransferases (SULT) and type 3 deiodinase in gestation. Consistent with high production rate of T4S and rT3S, there are high serum sulfated iodothyronine analogs, including T4S, T3S, rT3S, and 3,3’-T2S (T2S), in ovine and human fetal and preterm infants. Fetal TH metabolic pathways predict T2S as the major TH metabolite in the fetus. Since maternal T2S appears to be quantitatively derived from fetal T3 (the active TH), the amount of T2S in the maternal compartment correlates with fetal thyroid function in sheep. In humans, maternal serum contains high levels of radioimmunoassayable T2S; however, it displays as a peak adjacent to but unidentical to synthetic T2S on HLPC and we named it the W-Compound. Levels of W-Compound increase during pregnancy and peak as high as 20-fold to that of nonpregnant women. Maternal serum levels of W-Compound significantly correlate with fetal T4 and W-compound concentrations but not maternal serum T4 in euthyroid or hyperthyroid women, showing a distinct difference between fetal and maternal in TH metabolism. Fetal T2S is actively transferred to the mother via placenta and the quantity of T2S or its metabolite (W-Compound) in maternal compartment reflects fetal thyroid function. Thus, maternal serum W-Compound may be a biomarker for monitoring fetal thyroid function *in utero*, although more investigations are needed to determine if it can be used as an alternative strategy for screening/managing congenital hypothyroidism due to dysregulated thyroid hormone metabolism.

## Introduction:

The current screening program for congenital hypothyroidism (CHT) has allowed early treatment of this disorder and clearly improving long term outcomes [1–4]. However, despite the systematic screening and treatment of CHT, mild brain damages do occur [5, 6]. Since thyroid hormone (TH) is involved early first trimester fetal brain development including the neuronal cells [7–9], it is expected that developmental neuronal defects cannot be totally reversed postnatally. In developing mammals including humans, a deficiency or excess of TH in the developing brain during the fetal and neonatal periods can lead to morphological and functional abnormalities. Cretin is a serious form of congenital hypothyroidism (CHT), deficiency in TH in the newborn. These neonates suffer from not only impaired neurological function, but also stunted growth and physical deformities. This condition may occur in babies with a hypofunctioning thyroid gland. An estimated 15 to 20% of cases of CHT are inherited including gene mutations [1]. Many inherited cases are autosomal recessive but those with a mutation in the PAX8 gene or certain thyroid stimulating hormone receptor (TSHR) gene mutations have an autosomal dominant pattern of inheritance [3]. Other possible cause of fetal hypothyroidism is anti-thyroid medication treatment for maternal hyperthyroidism and lack of iodine during pregnancy. The incidence of babies born with CHT is 1 in 2,000 – 4,000 live birth in developed countries [3], representing a significant public health problem; this calls for more attention in better perinatal and neonatal care that needs new screening tools for fetal thyroid function and CHT. Our work in the sulfation pathway in mammalian fetal TH metabolism has obtained data that suggest the fetal-to-maternal transferred 3,3’-diiodothyronine sulfate (T_2_S)-like metabolite (W-Compound) can be used as novel marker for fetal thyroid function (10–12). The measurement of this compound in maternal serum and urine may serve as new marker for fetal thyroid function during *in utero* development.

### Current neonatal screening of thyroid function and CHT

Neonatal screening programs began detecting neonates with CHT over 45 years ago. At present, 38 million births yearly worldwide undergo screening for this disorder [3]. The screening program for CHT has allowed early treatment of this disorder and clearly improving long term outcomes [2–4]. However, despite the systematic screening and treatment of CHT, mild brain damages do occur [5, 6]. Since thyroid hormone (TH) is involved early first trimester fetal brain development including the proliferation, migration, and differentiation of neuronal cells [7–9], it is expected that developmental neuronal defects cannot be totally reversed postnatally. These irreversible changes can impact on child IQ, cognitive and motor measures [2, 5, 13–16]. Children affected may present reduced socio-educational achievement [17, 18], greater risk of autistic trait [14], and more ADHD (attention-deficit/hyperactivity disorder) symptoms [19]. Recently, it has been found that higher preconception maternal iodine intakes are associated with higher child IQ [20], indicating intervention before or during pregnancy may help the future outcome of children.

Unfortunately, the incidence of CHT in the United States showed a trend of increasing from ~ 1:4100 in 1987 to ~ 1:2400 in 2002 [21]. Similar increases (Table 1) were also observed in Australia [22], Italy [23], and Ireland [24]. Furthermore, some infants display a delayed thyroid stimulating hormone (TSH) rise that missed by neonatal screening [25]. Recent studies suggest that delayed TSH rise may be more common and more severe than previously recognized [26].

In addition, despite the U.S. being iodine sufficient for the general population, the U.S. dietary iodine intakes have decreased drastically since the 1970s, with deficiency reemerging in vulnerable groups such as women of reproductive age [26]. All these findings indicate that there is room for improvement in the current strategy with neonatal CHT screening. Further study of fetal thyroid hormone metabolism and function is warranted as these studies may provide alternative strategies for managing CHT to avert unwanted sequelae.

### What are the differences in thyroid hormone metabolism between fetus and adult?

**Figure 1:**
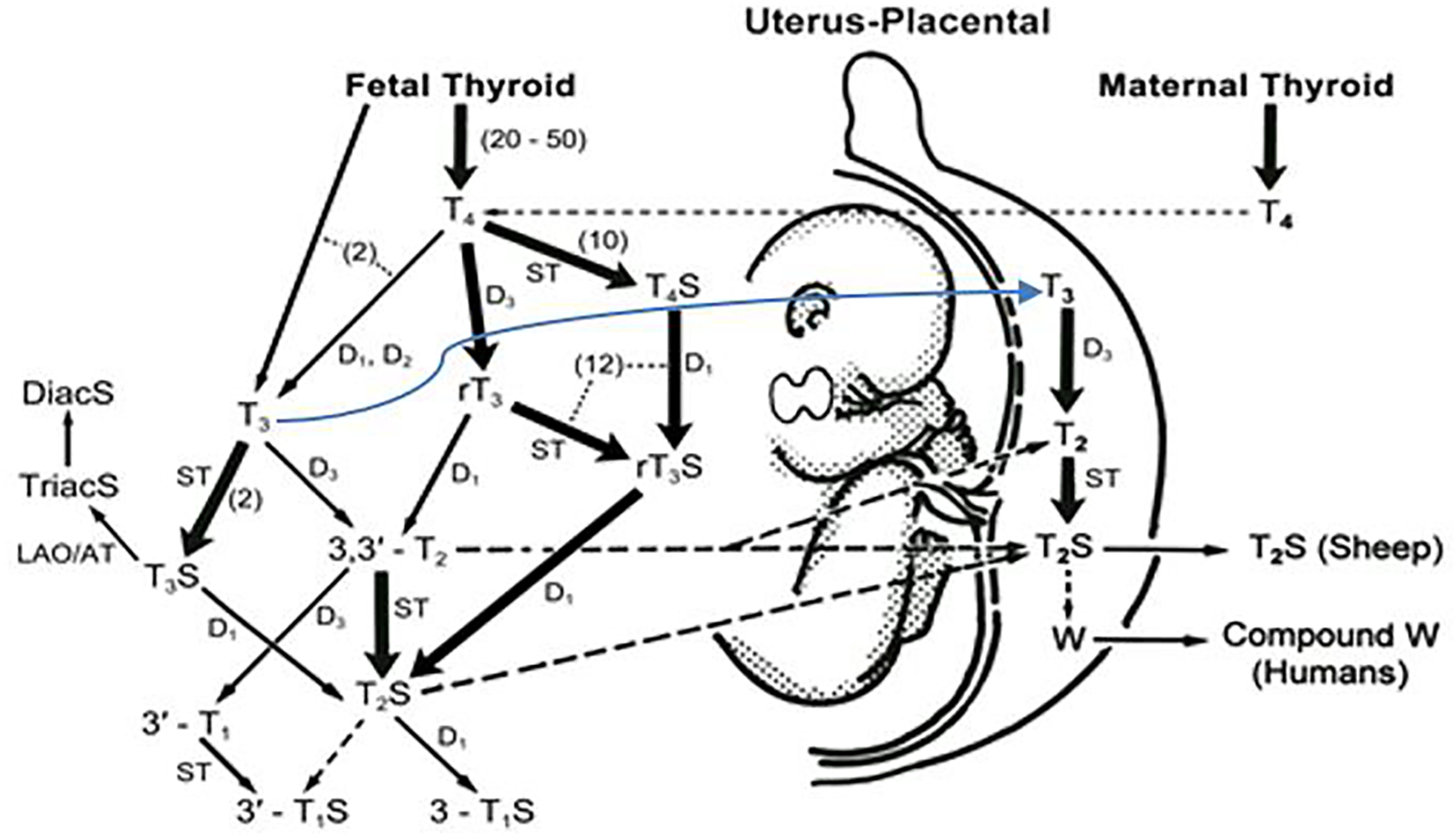
Postulated metabolic pathways for ovine fetal thyroid hormones (10, 30). Heavy solid lines indicate pathways that are more active in fetuses than in adults; thin solid lines, pathways that are less active in fetuses. The upper horizontal light dotted line depicts T_4_ of maternal origin moving to the fetal compartment in the first trimester, before the fetal thyroid begins functioning. The blue line indicates the transfer of fetal T_3_, through placenta D3 and ST to form T_2_S, into maternal compartment. Other broken lines represent unconfirmed pathways. Numbers in parentheses indicate published production rates (μg/kg/d). (D1, D2, and D3: type I, type II, and type III iodothyronine deiodinases; ST: iodothyronine sulfotransferases (SULT); LAO/AT: L-amino acid oxidase/aminotransferase; DiacS: sulfated 3,3’-diiodothyroacetic acid, TriacS sulfated 3,3’,5-triiodothyroacetic acid).

Our lab at University of California (Irvine) - Long Beach VA Medical Center, in collaboration with Professor D. A. Fisher at UCLA-Harbor General Medical Center, has found in mammalian fetuses that sulfoconjugation is the major pathway for TH metabolism (Figure. 1) [10, 27, 28].

Before the onset of active synthesis and release of TH, iodothyronines detected in the fetus clearly are maternal origin [15, 29]. This period is approximately the first 17 gestational days (d) in rats, 50d in sheep, and 90d in humans (Table 2 and Figure. 1, the upper horizontal light dotted line). The proposed scheme for ovine fetal iodothyronine metabolism in late gestation (near term) depicts the production rates for sulfoconjugated TH analogs (shown as numbers in parentheses along the thick arrows in Figure. 1).

A kinetic study using the steady state constant infusion method in sheep showed that the major pathways of TH metabolism in the fetus convert T_4_ to inactive metabolites, rT_3_, T_4_S, rT_3_S, and T_3_S, via sulfotransferase and D3 enzyme systems in late gestation [10, 27, 28]. The high production rate (μg/kg/d) of T_4_ sulfate (T_4_S) (Figure. 1) reflects the active activity of the sulfation pathway in the fetus [27,28]. The rT_3_S production rate likely represents both sulfation of rT_3_ and inner-ring deiodination of T_4_S.

Consistent with the high production rate of T_4_S and rT_3_S, we have shown high serum concentrations of sulfated iodothyronine analogs in ovine and human fetal and preterm infant sera. These include T_4_S, T_3_S, rT_3_S, and 3,3’-T_2_S (T_2_S) [27, 28, 31–40]. Elevated iodothyronine sulfoconjugates are also detectable in amphibians during metamorphosis [41].

Thus, in developing mammals, sulfoconjugation of iodothyronine is an important pathway, in particular, during late gestation when the hypophyseal-pituitary-thyroid system becomes more mature in precocial species including sheep and humans. As term approaches, fetal thyroid gland secretion increases progressively while the effects of TH in many peripheral tissues must be delayed to the postpartum period. D3 and SULTs may serve to moderate the circulating THs before parturition. In addition, the shunting of iodothyronine metabolites of fetal origin into maternal circulation, the fetal to maternal transfer, is also an important mechanism in keeping an optimal active TH level in the developmental fetus.

### The most common maternal circulating iodothyronine metabolite of fetal origin (-- the fetal to maternal transfer).

Thyroid hormone (TH) plays an important role in early fetal neurological maturation. Iodothyronines detected in the fetus before the onset of fetal thyroid function is of maternal origin. The maternal-fetal transfer of TH and their metabolites are apparently a two-way street. The high gradient between fetal and maternal serum concentrations of iodothyronine sulfates raises the possibility of significant fetal to maternal transfer of iodothyronine sulfoconjugates.

Sack et al. [42] reported that umbilical cord cutting, thus removing the lamb from placental D3 and transfer, triggers hypertriiodothyroninemia in the newborn lamb and that the postnatal T_3_ peak can be delayed until well after the TSH peak by delaying umbilical cord cutting. Santini et al. [43] reported that the placenta plays an important role in maintaining the low serum T_3_ in fetuses late in gestation. These findings suggest an important role of the placenta in fetal T_3_ metabolism, (Fig. 1, the blue line); it is possible that fetal-to-maternal transfer of the sulfated iodothyronines (via placenta) is one mechanism responsible for reducing serum T_3_ concentrations in the fetus. Increasing fetal-to-maternal transfer of iodothyronines occurs in late gestation.

The scheme shown in Fig. 1 also predicts 3,3’-T2S is the major thyroid hormone metabolite in the fetus. Intravenous infusion of radioiodine labeled T_3_ and T_4_ into near-term fetuses, demonstrated a rapid clearance of labeled T_3_ from fetal serum (disappearance T_1/2_ of 0.7 hours). Labeled T_2_S was identified as the major fetal iodothyronine metabolite in maternal urine [34]. Fetal T_3_ undergoes rapid inner-ring monodeiodination to 3,3’-T_2_ which is an excellent substrate for all known mammalian iodothyronine sulfotransferases [10]. The rapid sulfoconjugation of the hydroxyl group in the outer-ring of 3,3’-T_2_ forms a hydrophilic sulfated T_2_ (T_2_S) with enhanced permeability through placental membranes, facilitating the transfer of THs to maternal compartments. The T_2_S of fetal origin appears to be rapidly cleared from the maternal circulation via excretion in urine [44]. Fetal T_4_, on the other hand, disappears from the fetal circulation at a slower rate; a fast phase (T_1/2_=2.4 hours) in the first 3 hours followed by a slow phase (T_1/2_ = 17.5 hours). The major metabolites in fetal circulation after infusion of ^125^I-T_4_ were rT_3_ and T_3_ as well as their sulfates, T_4_S, rT_3_S and 3, 3’-T_2_S. Negligible amounts of T_3_S, roughly 0.7 – 1.2%, were also detected [44].

Similar to fetal T_3_ infusion, the most abundant metabolite found in maternal urine following radioactive T_4_ infusion is T_2_S. The T_4_ infusion study also confirms previous data in ovine fetuses [34, 35], indicating that the production of active thyroid hormone (T_3_) is less than the production of inactive products, rT3, T2S, rT3S and T3S [44].

T_3_ derived from T_4_ formed in the fetal circulation is converted to T_2_S, which is then transferred to the maternal compartment for deiodination/excretion. Recently, we have found sulfated [^125^I]-T_2_S was readily detected in the maternal compartment as the major metabolite of T_3_ following the perfusion of placenta with [^125^I]-T_3_ in guinea pig (12), suggesting that placental deiodinase and sulfotransferase may play an important role in fetal T_3_ homeostasis and in the fetal to maternal transfer of sulfated iodothyronine metabolites. This process would contribute to the low circulating T_3_ levels in the fetus. Since T_2_S appears to be quantitatively derived from circulating T_3_ (the active TH in the fetus), a significant increase or decrease in T_2_S in the maternal circulation would suggest hyper- or hypothyroidism in the fetus. In thyroidectomized sheep model, we found that 3,3’- T_2_S excretion in maternal urine reflects fetal thyroid function [45]. These data indicate clearly that maternal-fetal transfer of TH and its metabolites is a two-way street despite ovine placenta is less permeable as compared to rat and/or human (Table 2).

Furthermore, studies in rats have shown that 3,3’-T2 stimulates mitochondrial respiration in various tissues [46]. It is possible that a tight regulation of T_2_ concentration by sulfation and fetal-to-maternal transfer would have physiological value. Enhancing fetal-to-maternal transfer may protect the fetus from excessive mitochondrial thermogenesis stimulated by high fetal concentrations of T_2_. Another T_2_, i.e. 3,5-T_2_, was also shown to stimulate mitochondrial thermogenesis [46, 47]; however, its production rate is much lower in the fetus due to the inactive D1 (Figure 1).

### W-Compound, a T2S-immuno-crossreactive compound, ought to be considered as a fetal thyroid function marker.

**Figure 2:**
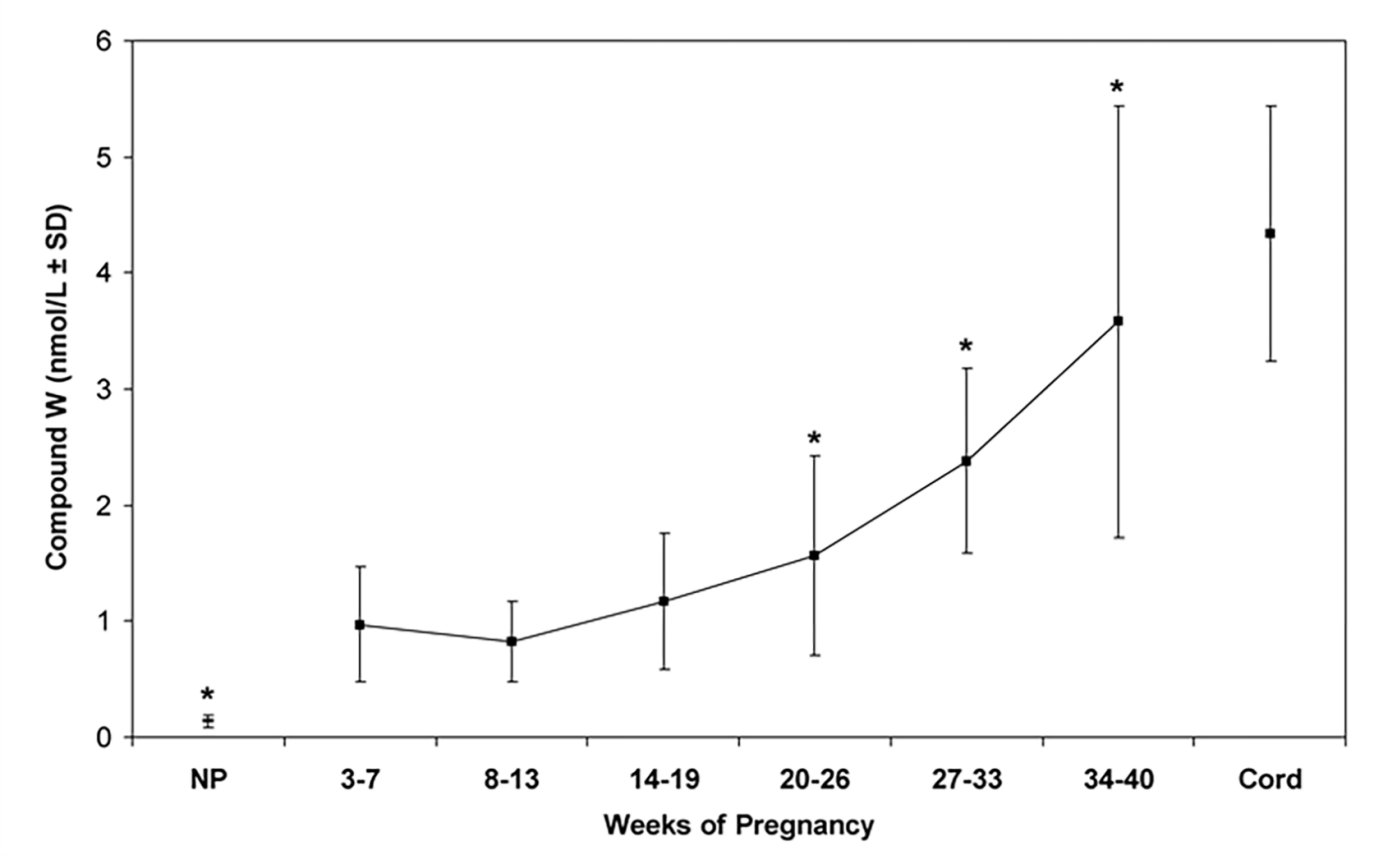
Changes of Compound W at different gestation periods. Normal values of T_2_S-crossreactive material (compound W) in serum from pregnant women, nonpregnant women (NP), and newborns. Vertical bars are mean ± 1 SD. * p < 0.05 cf. 3–7 weeks pregnancy.

**Figure 3:**
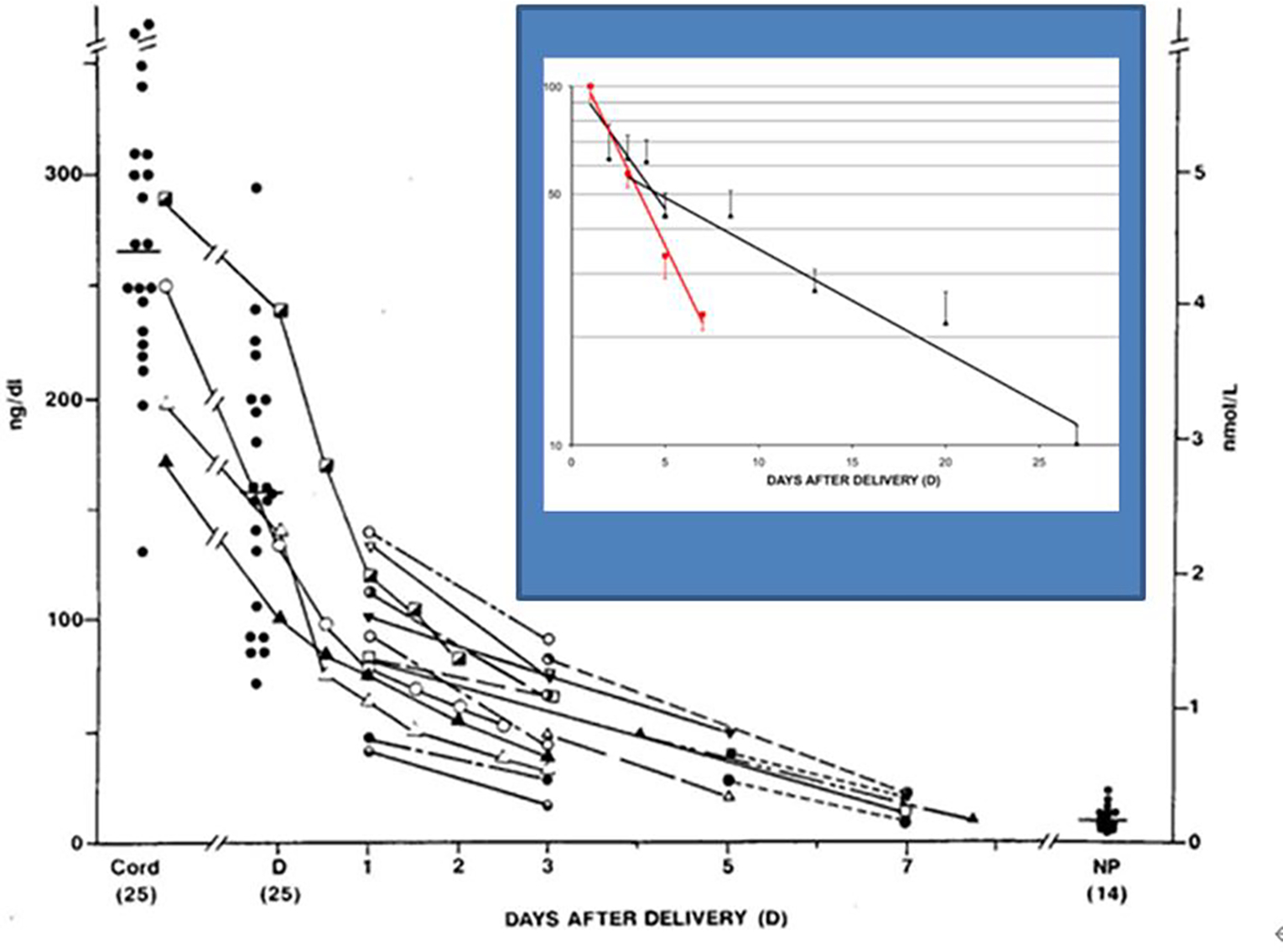
Concentrations of T_2_S and W-compound in cord serum of newborns and W-compound levels in maternal serum samples at the time of deliver (D). The connected lines represent serial measurements in the same patients (n = 18). T_2_S concentrations also were measured in 14 nonpregnant women (NP) for comparison. The percent reduction of levels in newborn and maternal groups in semi-log plot in the Insert: black lines are newborns; red line is post-partum mother. The closed red squares in vertical bars represent the mean (±SEM) and (n) represent the total number of samples studied at each time period in a total of 35 patients.

In humans, we have found high levels of radioimmunoassayable T_2_S in maternal serum [37, 39]; its levels increase with gestational age and peaked just prior to parturition. At delivery, a 20-fold increase in serum “T2S” is present compared to nonpregnant women (Figure 2) and “T2S” levels return to nonpregnant values in 7 to 10 days after delivery (Figure. 3). Serum levels were measured by a T_2_S-specific radioimmunoassay (RIA) in 60 serum samples from newborns with hyperbilirubinemia, age 1 to 30 days. It is found that radioimmunoassayable T_2_S is cleared at similar rates in newborn as in postpartum maternal sera. This is consistent with the hypothesis that this “T2S” is produced in the placenta [46] (Figure 3).

**Figure 4:**
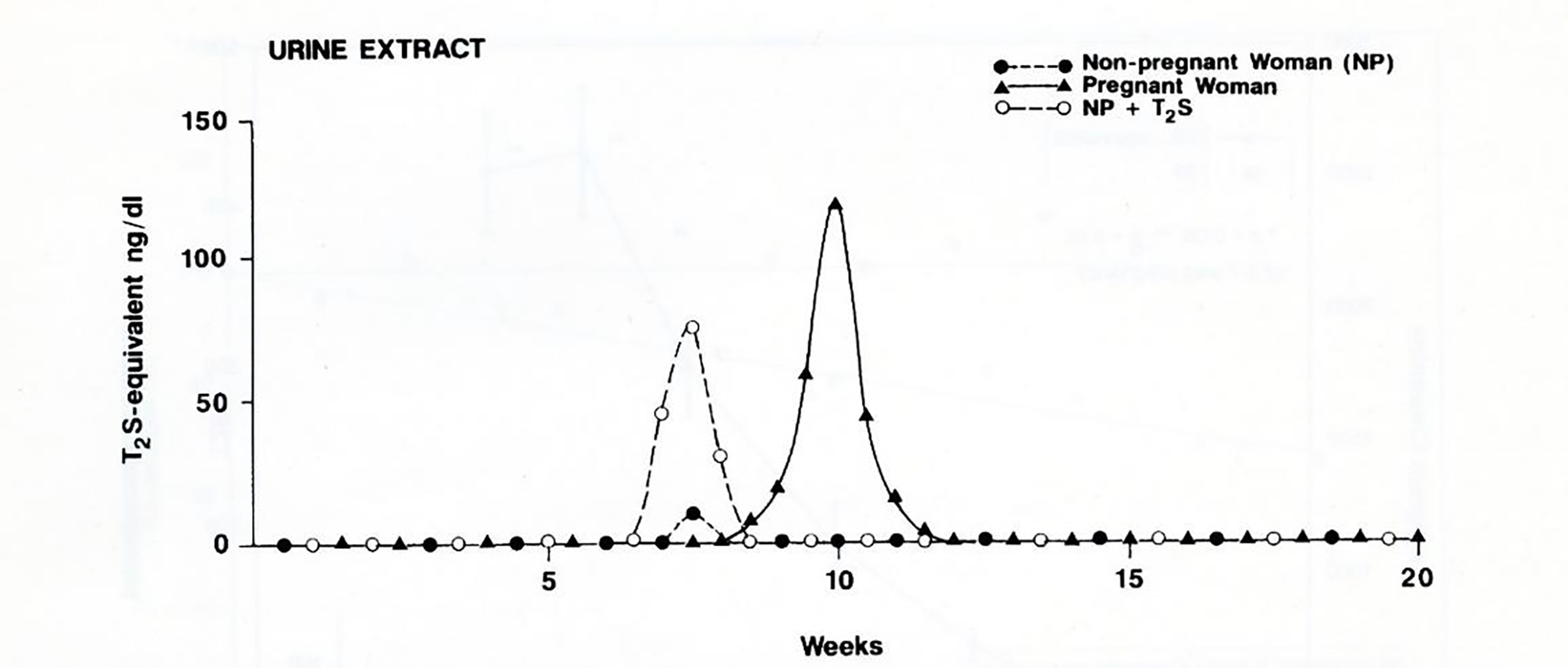
Elution patterns of W-compound and T_2_S which were identified by a sensitive RIA. Samples were eluted from HPLC isocratically with a mixture of acetonitrile and 0.02 mol/L ammonium acetate, pH 4.0 (22:78 vol/vol).

On closer examination, the radioimmunoassayable “T2S” did not cochromatography with synthetic T_2_S by HPLC [39], (Figure 4). Over 40 known synthetic thyroid hormone analogs that were examined, none was found to be identical to the serum T_2_S-like material in pregnant women [49]. Thus, the name W-Compound was given. It is postulated that W-Compound is a side-chain modification of T_2_S, which cross-reacts with T_2_S antibody but is slightly more hydrophobic than T_2_S. Consistent with being an analogue of iodothyronine, we found high level of iodine content in highly purified W-Compound preparation analyzed by a Triple Quadrupole ICP-MS (Inductively Coupled Plasma Mass Spectrum) [50].

**Figure 5:**
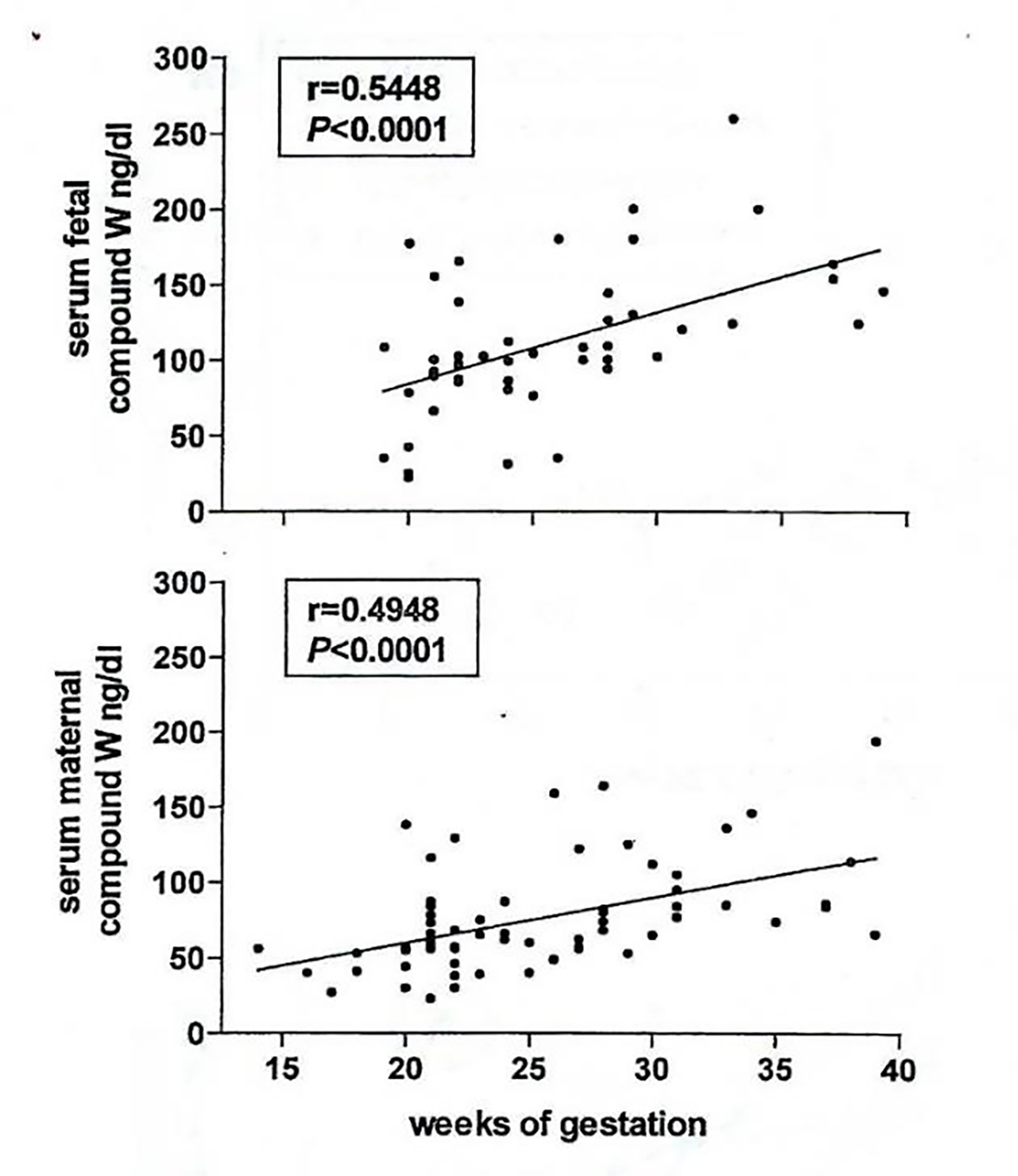
W-compound levels in 49 fetal and 64 maternal sera correlation with the weeks of gestation.

**Figure 6:**
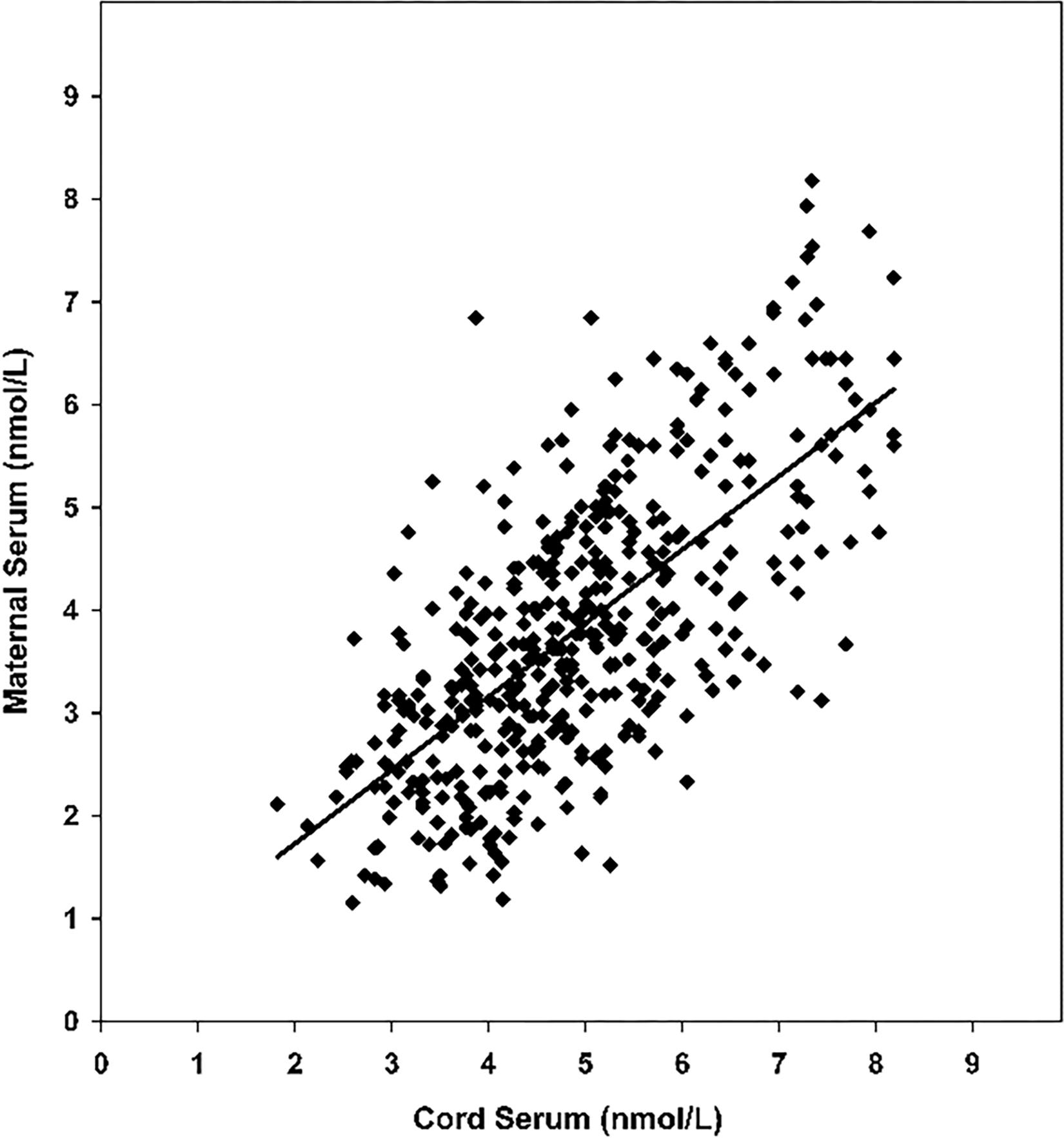
Levels of T_2_S-crossreactive material, W-compound, in paired maternal and cord serum at term. The solid line is the trend-line from lineal regression analysis for the correlation (n = 436, R = 0.686).

In normal pregnancy, both maternal and fetal W-Compound levels increase progressively with a significant direct correlation (p<0.001, in both mothers and fetuses) [51], (Figure 5). In addition, in 436 paired cord and maternal sera obtained from women at delivery, there is a highly significant correlation between the concentrations of Compound W in newborn cord and maternal sera (p<0.01) [49], (Figure 6).

**Figure 7:**
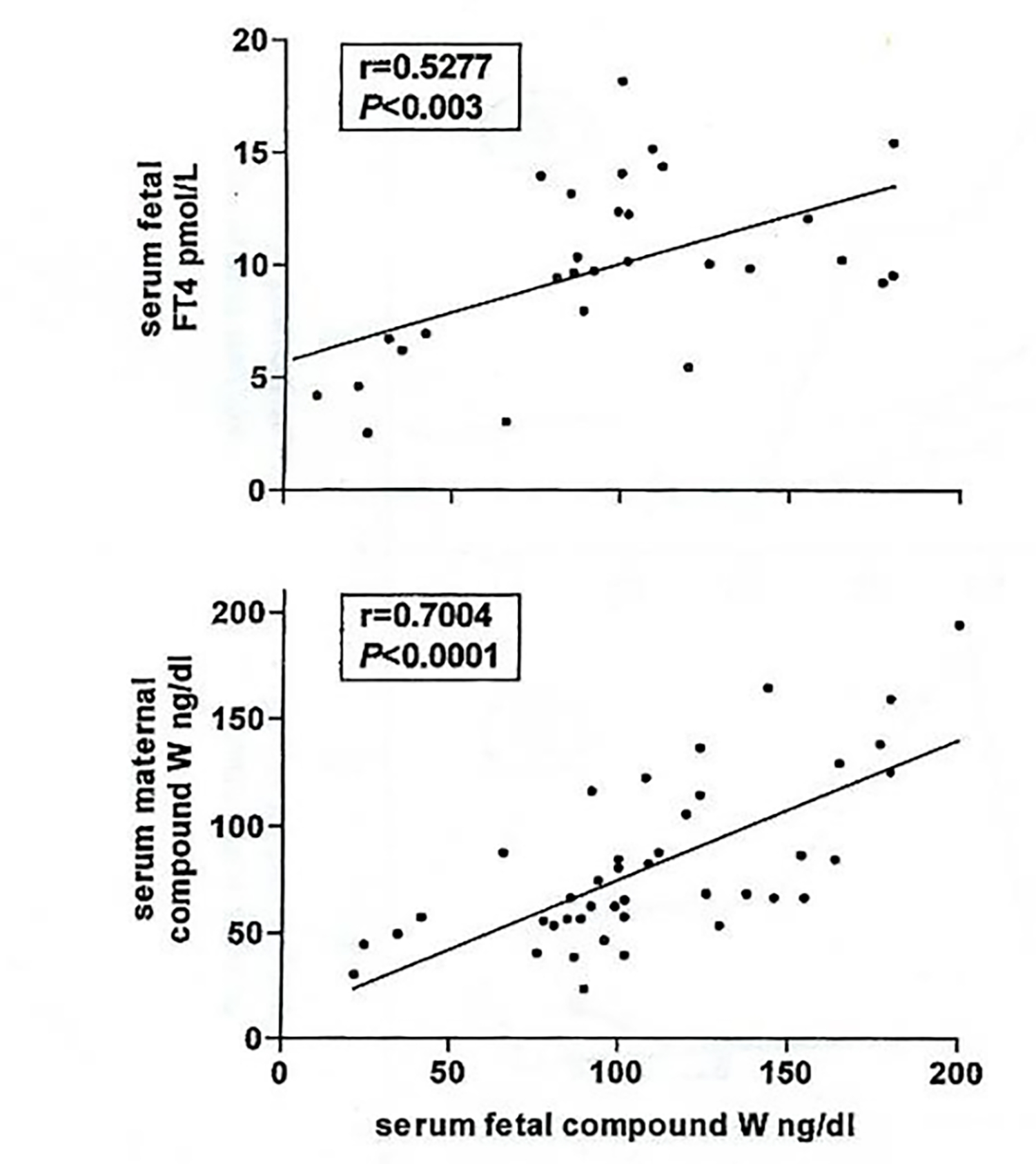
W-Compound levels in fetal serum correlation with serum fetal FT4 (n=29) and maternal W-compound (n=42).

A significant positive correlation is also observed between fetal serum concentrations of W-Compound and fetal T_4_ (p<0.003) and between maternal and fetal W-Compound concentrtions (p<0.0001) [51], (Figure. 7). However, no significant correlations were observed between maternal serum W-Compound and maternal serum T_4_ in euthyroid or hyperthyroid women. These data strongly suggest the fetal origin of W-Compound.

To further explore the possible origin of W-Compound, the serum concentrations of sulfated iodothyronines from cord arterial and venous blood samples were compared [49]. There were no significant differences between the mean T_3_S, T_4_S, or reverse-T_3_S concentrations of arterial and venous serum samples. However, the venous concentration of the T_2_S-equivalent material was higher than that in arterial blood in seven of the paired samples and lower in two. The mean “corrected” concentration of W-Compound in nine pairs of cord sera was found to be significantly higher in venous than arterial blood samples suggesting the fetal origin of W [49]. In addition, the mean of the maternal serum concentrations of T_2_S-reactive material was significantly lower than that of the paired cord serum concentrations. The rapid disappearance of W-Compound from maternal blood immediately after delivery supports this hypothesis [39], (Figure 3). A similar disappearance slope of serum W-Compound was also found in newborn infants [48], (Figure. 3, insert). These findings support the postulation that W-Compound is produced in placenta with iodothyronine precursor of fetal origin.

### The Measurement of W-Compound: a technical consideration.

The original method for the measurement of W-Compound involves the use of RIA which was developed by Wu et al. [39]. Radioimmunoassay, in general, is not convenient to most clinical laboratories due to the involvement of using a radioisotope I^125^.

In a recent study, we have applied a highly sensitive and rapid homogeneous time-resolved fluorescence immunoassay to establish an indirect competitive W-Compound quantitative detection method called AlphaLisa (ICW-AlphaLisa), to measure the levels of W-Compound in maternal serum during pregnancy [52]. We developed specific polyclonal antibodies against W-Compound [a 3,3’-diiodothyronine sulfate (T_2_S) immuno-crossreactive material] and established an ICW quantitative detection method using AlphaLISA. In this method, photosensitive particles (donor beads) were coated with purified W-Compound or T_2_S and rabbit anti- W-Compound antibody, followed by incubation with biotinylated goat anti-rabbit antibody. This constitutes a detection system with streptavidin-coated acceptor particle. We have optimized the test conditions and evaluated the detection performance. The sensitivity of the method was 5 pg/ml in a detection range of 5–10,000 pg/ml. The intraassay coefficient of variation averages <10% with stable reproducibility. The ICW-AlphaLISA shows good stability and high sensitivity and can measure a wide range of W-compound levels in extracts of maternal serum samples. This may have clinical application to screen congenital hypothyroidism in utero [52].

Brominated flame retardants (BFRs) have been recently shown to disrupt TH homeostasis through multiple mechanisms (53), including inhibition of enzymes that regulate intracellular levels of THs, such as sulfotransferases (SULTs). As discussed in the present review, the placenta plays a critical role in expressing D3 and SULTs to prevent the developing fetuses from exposure to high level of active thyroid hormone T_3_, which are needed immediately after birth. The adverse effect of BFRs is concerning, given that disruption of TH regulation within the placenta could potentially harm the developing fetus [28, 29]. Iodothyronines and their sulfoconjugates in these studies were measured by liquid chromatography-tandem mass spectrometry (LC/MS-MS) [54, 55]. Even though the claim was made that this method was comparable to RIA, however, the sensitivities to detect for 3,3’-T2 and T2S were difficult to judge. Nevertheless, the lowest concentrations of standards used to optimize and calibrate the LC/MS-MS varied between 1–10 ng/ml that was much higher than the serum levels of 3,3’-T2 and T2S in physiological states [37, 53– 56].

## Conclusions

Sulfoconjugation is a major metabolic pathway for thyroid hormone in developing mammals. The significant rise of sulfated iodothyronines in fetal compartments raises the possibilities that remarkable fetal to maternal transfer of the TH sulfoconjugates may occur throughout the second and third trimester in humans. This transfer may be a novel mechanism to maintain low T_3_ states or regulate serum 3,3’-T_2_, a thermogenic hormone, that is important for normal tissue maturity. The possibility that the transferred iodothyronine sulfate, especially 3,3’-T2S and its metabolite, may serve as a biomarker of fetal thyroid function needs to be further explored. Because the placenta plays a critical role in expressing D_3_ and SULTs to prevent the developing fetuses from exposure to high level of active thyroid hormone T_3_, which is needs immediately after birth. To this end, the non-isotopic method we developed [49] provides a very valuable means to facilitate future studies on W-Compound as a fetal thyroid function biomarker. Because disruption of TH regulation within the placenta could potentially harm the developing fetus [28], further studies are warranted to explore the possibility of the maternal serum or urine levels of W-Compound as a biomarker for BFR toxicity.

## Figures and Tables

**Figure 8:**
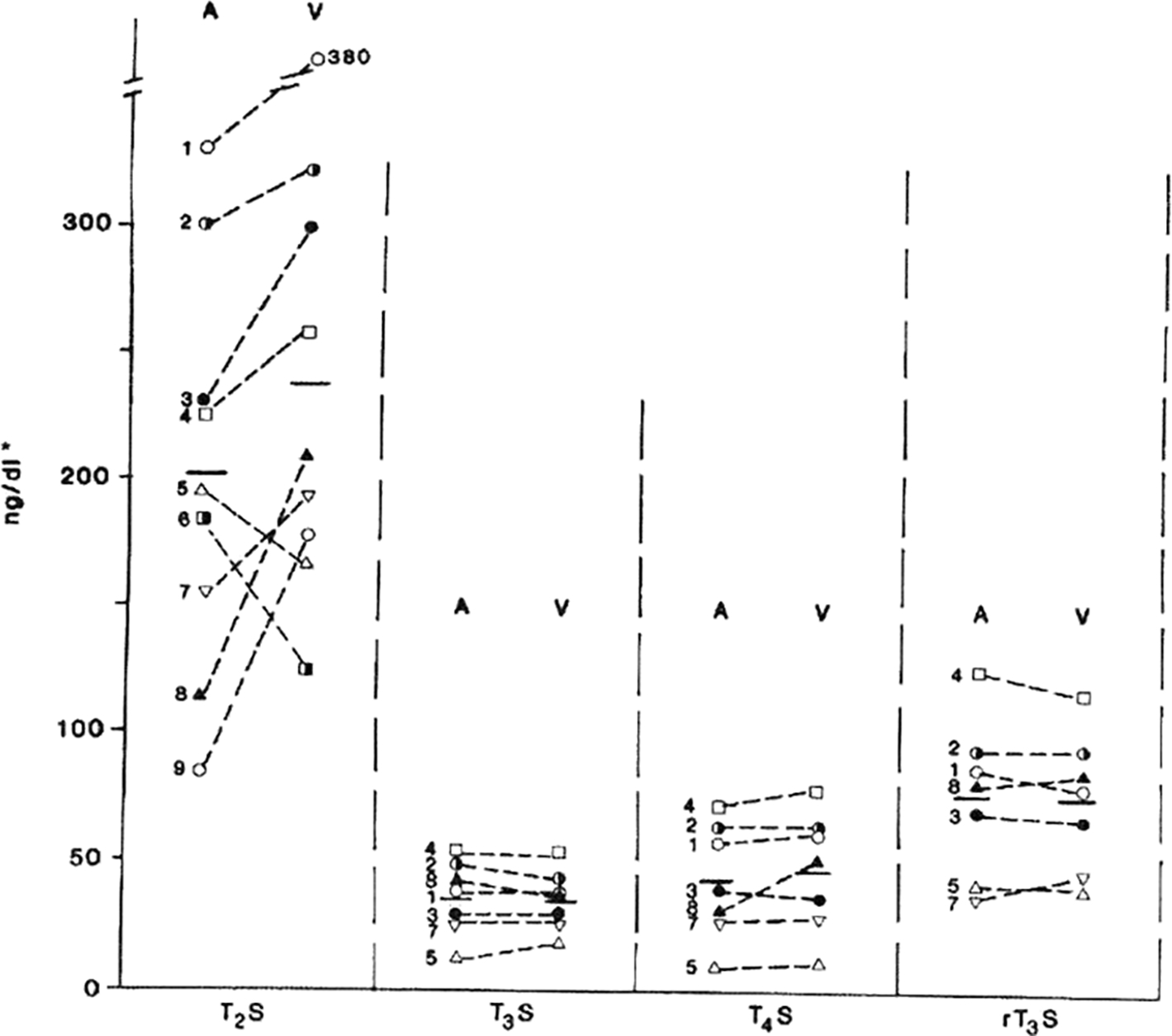
Sulfated iodothyronine (or the equivalence in T2S) levels in arterial (A) and venous (V) cord serum. Horizontal bars indicate the mean. * For conversion to nmol/L T2S, multiply by 0.0165; nmol/L T3S and rT3S, multiply by 0.0156; nmol/L T4S, multiply by 0.0148.

**Table 1:** Incidence of congenital hypothyroidism (Per 1000 Live Births) in developed countries.

Nations/Time	1980–1989	1990–1999	2000–2009	2010–2019	Note
U.S.A.	0.24		0.42		Ref. 21
(Western) Australia	0.17	0.35			Ref. 22
Italy		0.33	0.51		Ref. 23
Ireland	0.27	0.38	0.50	0.75	Ref. 24

**Table 2:** Comparison of sheep, rat and humans in the study of fetal-to -maternal transfer of iodothyronines in pregnancy.

	Sheep	Human	Rat

**Length of Gestation**	150 d	280 d	21 d

**Species**	precocial	precocial	altricial

**Thyroid Function at birth**	mature (similar to humans)	mature	immature (2nd trimester to human)

**CNS Development at birth**	mature	intermediate	immature

**Placenta: type:**	epitheliochorial	haemomonochorial	haemotrichorial
**origin:**	maternal and fetal	fetal only	fetal only
**layers:**	6	3	4

**Placental permeability to TH (vs. human)**	less permeable	----	more permeable

**Animal model to study placenta in late gestation**	yes	----	no

## Abbreviations:

ADHD: Attention Deficit/Hyperactivity Disorder
BFR: Brominated flame retardants
CHT: Congenital Hypothyroidism
D1, D2, and D3: Type I, Type II, And Type III Iodothyronine Deiodinase
DiacS: Sulfated 3,3’-Diiodothyroacetic Acid
LAO/AT: L-Amino Acid Oxidase/Aminotransferase
SULT or ST: Sulfotransferases
T1, T2 and T3: Mono-, Di-, and Tri-iodothyronine
T4: Thyroxine
T4S, T3S, rT3S, T2S and T1S: Sulfated T_4_, T_3_, rT_3_, T_2_ and T_1_
TH: Thyroid Hormone
TriacS: Sulfated 3,3’,5-Triiodothyroacetic Acid
TSH: Thyroid Stimulating Hormone
TSHR: TSH Receptor
